# Late-presenting posterior urethral valve with secondary polypoid urethritis in an adult male presenting with recurrent urinary retention: A case report

**DOI:** 10.1016/j.eucr.2026.103648

**Published:** 2026-10-26

**Authors:** Johannes Cansius Prihadi, Darren Wijaya, Alvin Chandra, Dyonesia Ary

**Affiliations:** a Division of Clinical Medicine, Faculty of Medicine and Nutrition, IPB University, Bogor, West Java, Indonesia; b Department of Surgery, Sub Urology, St. Carolus Hospital, Jakarta, Indonesia; c Faculty of Medicine and Health Sciences at Atma Jaya Catholic University of Indonesia, Jakarta, Indonesia; d Department of Anatomical Pathology at Atma Jaya Catholic University of Indonesia, Jakarta, Indonesia

**Keywords:** Posterior urethral valve, Urinary retention, Polypoid urethritis

## Abstract

Posterior urethral valve (PUV) is a rare congenital cause of bladder outlet obstruction that is infrequently diagnosed in adults. We report a rare case of late-presenting posterior urethral valve with secondary polypoid urethritis in a 32-year-old man presenting with recurrent urinary retention. Ultrasonography was inconclusive, whereas cystoscopy identified an obstructing posterior urethral valve, which was treated by transurethral endoscopic resection. Histopathology demonstrated secondary reactive polypoid urethritis without malignancy. The patient remained symptom-free at one-month follow-up. Adult PUV should be considered in recurrent urinary retention with inconclusive investigations, and early cystoscopy may prevent delayed diagnosis and treatment.

## Highlights

• Adult PUV may be overlooked despite inconclusive routine investigations.
• A failed trial without a catheter (TWOC) should prompt early cystoscopic evaluation.
• Cystoscopy provided both definitive diagnosis and treatment.
• Prolonged catheterization after delayed diagnosis may induce reactive changes.

## 1 Introduction

Posterior urethral valves are a rare congenital disorder affecting the male urinary tract, characterized by abnormal membranous folds in the posterior urethra. This condition can lead to urinary tract obstruction, resulting in a spectrum of clinical manifestations ranging from delayed voiding to severe urinary retention.^1^ Posterior urethral valve (PUV) is a congenital obstructive defect of the male urethra with an incidence of 1/8000 to 1/25,000 live births. PUV is typically diagnosed in early life and cases identified in adulthood remain uncommon.^2^ Approximately three-quarters of PUV cases are identified prenatally through antenatal ultrasonography^3^ or during early infancy, when boys are evaluated for hydronephrosis or voiding dysfunction.^4^

In contrast, presentation beyond childhood is distinctly uncommon, and diagnosis in adulthood is considered rare, with only isolated cases described in the literature.^3^, ^5^ Adult patients with PUV may present with a broad spectrum of nonspecific lower urinary tract symptoms ranging from obstructive voiding complaints to postejaculatory dysuria, which often overlap with more common urologic conditions such as cystitis, urethral stricture, or prostatic pathology.^2^, ^3^ This symptom overlap frequently delays recognition, as clinicians may not initially suspect a congenital urethral anomaly in an adult without prior urologic history.^3^, ^5^ Some authors have suggested that later age at presentation may reflect a comparatively milder degree of obstruction, allowing patients to remain undiagnosed for years despite ongoing lower urinary tract dysfunction.^5^, ^6^ Conditions such as benign prostatic hyperplasia, urethral stricture, prostatitis, urethritis, and sphincter–bladder dyssynergia are frequently superimposed on late-presenting PUV, thus making correct diagnosis difficult.^6^

Given the rarity of adult-onset PUV and its potential to mimic more common causes of urinary retention, heightened clinical awareness is essential to avoid diagnostic delay and to guide appropriate endoscopic management. We report a case of an adult male presenting with an obstructing posterior urethral polypoid lesion, ultimately diagnosed as an adult manifestation of posterior urethral valve disease with associated inflammatory polypoid changes, to contribute to the limited existing literature on this uncommon presentation.

## 2 Case illustration

A 32-year-old man presented with a two-month history of recurrent urinary retention characterized by progressive difficulty initiating micturition, suprapubic fullness, dysuria, and incomplete bladder emptying. Despite a strong urge to void, only minimal urinary dribbling occurred. The patient had no significant medical or urological history, recurrent urinary tract infections, urethral trauma, or previous urological procedures or surgery. An indwelling urethral catheter was inserted for bladder decompression and maintained for approximately one month. Following symptomatic improvement, a Trial Without Catheter (TWOC) was attempted; however, acute urinary retention recurred within 24 hours, necessitating emergency catheter reinsertion. Due to recurrent urinary retention after a failed TWOC and unremarkable initial imaging, diagnostic cystoscopy was undertaken to evaluate a suspected anatomical bladder outlet obstruction.

The patient had no significant medical or urological history and denied smoking, alcohol consumption, recreational drug use, high-risk sexual behavior, or prior sexually transmitted infections. No abnormalities were identified on physical examination, including external genital examination. Laboratory investigations revealed mild anemia, whereas the remaining hematological, renal, and liver function parameters were normal. Urinalysis was unavailable. Ultrasonography showed normal kidneys and prostate without structural abnormalities. Mild bladder wall thickening was observed, and the initial radiological impression was suspected cystitis.

Cystoscopy identified a congenital posterior urethral valve presenting as a obstructive lesion arising from the posterolateral of posterior urethra and extending toward the bladder neck, causing significant bladder outlet obstruction. Complete transurethral endoscopic resection was performed (Fig. 1). The excised lesion was submitted for histopathological examination as multiple irregular tan-brown, firm tissue fragments measuring approximately 5 cc in aggregate. Histopathological examination revealed polypoid urethritis characterized by eroded urothelium with edematous fibrovascular stroma and mixed acute and chronic inflammatory cell infiltration, without evidence of malignancy (Fig. 2). Following complete endoscopic resection, the patient recovered without postoperative complications and was managed with analgesic and antibiotic therapy. The Foley catheter was removed on postoperative day 3, and the patient successfully passed a TWOC with minimal postoperative pain. At the one-month follow-up, the patient reported complete resolution of lower urinary tract symptoms, restoration of a normal urinary stream, and no recurrence of urinary retention.

**Fig. 1:**
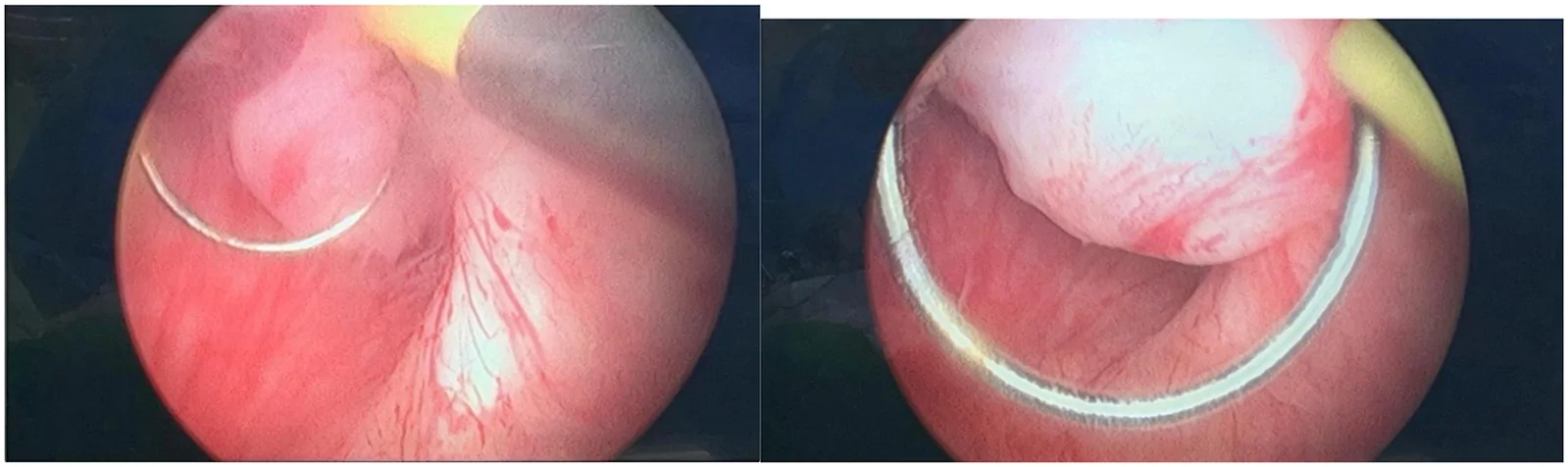
Cystoscopic appearance of the obstructing posterior urethral valve before resection. (a) Pedunculated configuration of the obstructing valve arising from the posterior urethra. (b) Extension of the obstructing lesion toward the bladder neck.

**Fig. 2:**
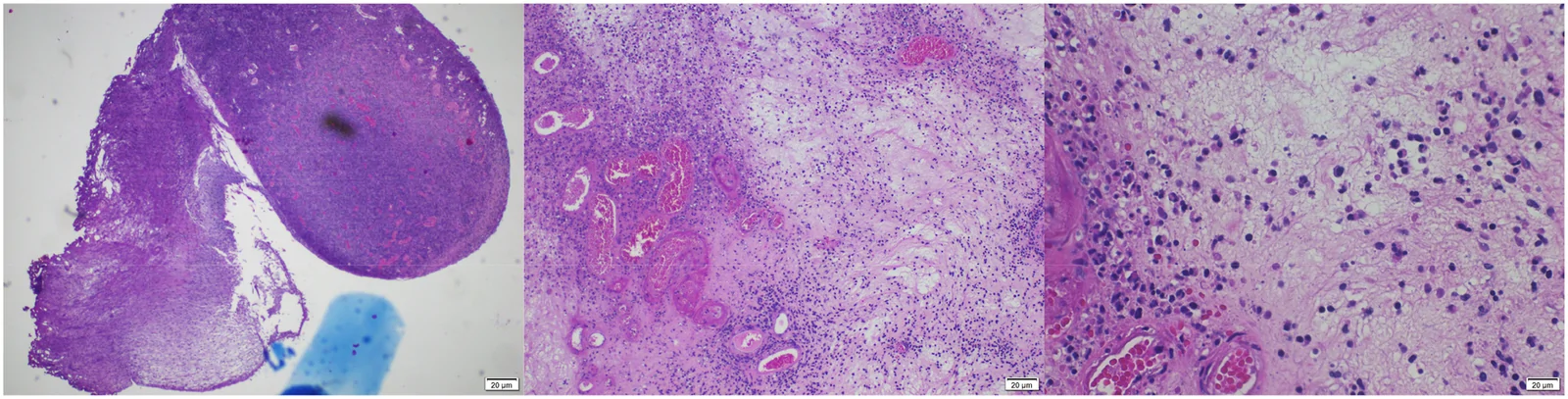
Histopathological findings of polypoid urethritis. (a) Polypoid appearance with inflammatory cell infiltration. (b) Extensive urothelial erosion with edematous fibrovascular stroma and fibrinoid necrosis. (c) Mixed acute and chronic inflammatory cell infiltration.

## 3 Discussion

Late-presenting PUV is a rare but important cause of recurrent urinary retention in adults, frequently posing a diagnostic challenge because routine investigations may fail to identify the underlying anatomical obstruction. PUV is the most common cause of congenital bladder outlet obstruction in male infants, with an estimated incidence ranging from 1 in 8000 to 1 in 25,000 live births.^2^ Embryologically, it is believed to result from abnormal proximal urethral development, possibly related to persistent Wolffian duct insertion into the urogenital sinus or a congenital obstructing posterior urethral membrane.^1^, ^7^ The severity of obstruction varies considerably, allowing some patients to remain clinically compensated for years before presenting later in life with nonspecific lower urinary tract symptoms or recurrent urinary retention rather than the typical pediatric manifestations.^2^, ^8^ This atypical clinical course was well illustrated in our patient, whose congenital obstruction remained undiagnosed until adulthood despite recurrent urinary retention, highlighting the importance of maintaining a high index of suspicion for congenital etiologies even in adult patients.

The delayed diagnosis in our patient illustrates this diagnostic dilemma. Despite recurrent urinary retention, renal function remained preserved and ultrasonography demonstrated only mild bladder wall thickening without hydronephrosis or prostatic abnormalities. As no definitive anatomical cause of bladder outlet obstruction was identified on ultrasonography, the initial clinical suspicion for congenital posterior urethral valve was low. Persistent urinary retention following a failed TWOC ultimately necessitated cystoscopic evaluation. Although voiding cystourethrography (VCUG) is the definitive imaging modality for diagnosing PUV,^5^ in our case, we made the diagnosis and the treatment simultaneously during cystourethroscopy, thus we did not perform VCUG.

This case presents the coexistence of PUV and secondary polypoid urethritis, a reactive inflammatory lesion arising from chronic urothelial irritation rather than a true neoplasm.^9^ In our patient, prolonged urethral catheterization with repeated catheter reinsertion preceded surgical management and histopathological examination demonstrated by urothelial erosion, stromal edema, fibrinoid necrosis, and mixed acute and chronic inflammatory cell infiltration. Although a direct causal relationship cannot be established from a single case, the temporal sequence and pathological findings suggest that prolonged mechanical irritation may have contributed to the secondary inflammatory changes observed.^9^ This observation provides a plausible explanation for the discrepancy between the endoscopic diagnosis of PUV and the histopathological diagnosis of polypoid urethritis while emphasizing the importance of interpreting pathological findings within the appropriate clinical context.

From a clinical perspective, this case reinforces that PUV or other congenital bladder outlet obstruction should remain part of the differential diagnosis in adult men presenting with unexplained or recurrent urinary retention.^2^ Because routine imaging may fail to demonstrate an anatomical cause, recurrent retention following a failed TWOC should prompt early cystoscopic evaluation rather than repeated conservative management. Furthermore, histopathological examination remains essential, not only to exclude malignancy but also to recognize reactive inflammatory changes that may otherwise be misinterpreted as a discrete pathological entity.

To our knowledge, reports describing late-presenting adult PUV accompanied by secondary reactive polypoid urethritis following prolonged urethral catheterization remain exceedingly scarce. Nevertheless, this report has several limitations, including the absence of preoperative voiding cystourethrography and uroflowmetry, the inability to definitively distinguish a true congenital valve from an acquired obstructive lesion on histopathological grounds alone, the relatively short follow-up period, and the limitation of a single case report in establishing a causal relationship between prolonged catheterization and the observed inflammatory changes.

## 4 Conclusion

This case underscores that PUV, although rare in adulthood, should remain part of the differential diagnosis in men with recurrent urinary retention unexplained by initial imaging. Cystoscopy proved essential for diagnosis, and complete endoscopic resection achieved full symptomatic resolution. The coexisting histopathological finding highlights the importance of correlating endoscopic and pathological findings within the clinical context, particularly in patients with prolonged urethral catheterization.

## CRediT authorship contribution statement

**Johannes Cansius Prihadi:** Writing – original draft, Visualization, Supervision, Methodology, Investigation, Data curation, Conceptualization. **Darren Wijaya:** Writing – original draft, Validation, Resources, Investigation, Conceptualization. **Alvin Chandra:** Writing – review & editing, Validation, Methodology. **Dyonesia Ary:** Writing – review & editing, Validation, Supervision, Methodology.

## Ethics declaration

Written informed consent to take part in the study and to publish the article has been obtained from all participants or their legal representatives. The privacy rights of participants have been observed.

This study included organ or tissue donors. This study includes human biological material and consent was obtained by donors, or their next of kin or legal representatives, for use in this study and for publication of the article. The samples used in this research were not sourced from executed prisoners or prisoners of conscience.

This study was performed in compliance with relevant laws, regulatory frameworks and guidelines where the research took place. Ethics committee approval was not required under relevant laws and institutional guidelines. Ethics committee approval was exempt for this case report in accordance with institutional guidelines and applicable local regulations.

## Declaration of competing interest

The authors declare that they have no known competing financial interests or personal relationships that could have appeared to influence the work reported in this paper.
